# Predictive value of liver enzymes in long-term prognosis of hepatic Wilson disease: results from the Wilson AEEH registry

**DOI:** 10.1186/s13023-025-03821-1

**Published:** 2025-06-07

**Authors:** Marina Berenguer, Luis García-Villarreal, Antonio Olveira, Esther Mollina Pérez, José María Moreno Planas, Marta Romero-Gutiérrez, José María Pinazo Bandera, Helena Masnou Ridaura, Paula Iruzubieta, María Luisa González Diéguez, Javier Ampuero, José Ramón Fernández Ramos, Carolina Muñoz, Ana Arencibia Almeida, Sara Lorente, Manuel Delgado Blanco, Diego Burgos Santamaría, Mònica Pons Delgado, Alba Cachero, Manuel Hernández Guerra, Judith Gómez Camarero, Sergio Gil Rojas, María Lázaro Ríos, Isabel Carmona Soria, Gemma Carrión, Ariadna Bono, Anna Miralpeix, Pablo Alonso Castellano, Zoe Mariño, Registro Wilson-AEEH

**Affiliations:** 1 Hepatology and Liver Transplantation Unit, Hospital Universitari I Politècnic La Fe, IISLaFe, Ciberehd and Valencia University, Avda Fernando Abril Martorell no 106, 46026 Valencia, Spain; 2 IUIBS Universidad Las Palmas Gran Canaria. Servicio de Digestivo, CHUIMI, Las Palmas de Gran Canaria, Spain; 3 Hospital Universitario La Paz, Madrid, Spain; 4 Hospital Clínico de Santiago, Santiago de Compostela, Spain; 5 Servicio de Aparato Digestivo, Facultad de Medicina, Complejo Hospitalario Universitario de Albacete, Universidad de Castilla La Mancha, Ciudad Real, Spain; 6 Hospital Universitario de Toledo, Toledo, Spain; 7 Unidad de Hepatología, Unidad de Gestión Clínica de Aparato Digestivo, Hospital Universitario Virgen de La Victoria, Instituto de Investigación Biomédica de Málaga-Plataforma Bionand, Málaga, Spain; 8 Unitat Hepatologia, Servei Aparell Digestiu, Hospital Germans Trias I Pujol, Badalona, Spain; 9 Hospital Universitario Marqués de Valdecilla, Santander, Spain; 10 Hospital Universitario Central de Asturias, Oviedo, Spain; 11 Hospital Universitario Virgen del Rocío, Seville, Spain; 12 Hospital Universitario de Cruces, Barakaldo, Spain; 13 Hospital Universitario, 12 de Octubre, Madrid, Spain; 14 Hospital Universitario Nuestra Señora de La Candelaria, Santa Cruz de Tenerife, Spain; 15 Unidad de Hepatología y Trasplante Hepático, Hospital Clínico Lozano Blesa de Zaragoza, IISS Aragón, Zaragoza, Spain; 16 Hospital Universitario A Coruña, A Coruña, Spain; 17 Hospital Ramón y Cajal, Madrid, Spain; 18 Servicio de Hepatología, Hospital Vall d’Hebron, Universitat Autònoma Barcelona, CIBERehd, Barcelona, Spain; 19 Hospital Universitari de Bellvitge, Hospitalet de Llobregat, Spain; 20 Hospital Universitario de Canarias, Santa Cruz Tenerife, Spain; 21 Hospital Universitario de Burgos, Burgos, Spain; 22 Hospital Universitario Virgen de La Luz, Cuenca, Spain; 23 Hospital Universitario Miguel Servet, Zaragoza, Spain; 24 Hospital Virgen Macarena, Seville, Spain; 25 Hospital Universitario Infanta Leonor, Madrid, Spain; 26 Hospital Universitari I Politècnic La Fe, Valencia, Spain; 27 Liver Unit, Hospital ClínicCIBERehd, IDIBAPS, ERN-RARE Liver, Universitat de Barcelona, Barcelona, Spain

**Keywords:** Wilson disease, Spanish Wilson registry, Transaminases, Cirrhosis, Fibrosis progression

## Abstract

**Background and Aims:**

Monitoring Wilson disease (WD) is challenging due to its variable presentation and the absence of reliable biomarkers. This study aims to assess the predictive value of liver enzymes, particularly transaminases, on long-term outcomes in patients with hepatic WD using data from the Spanish Wilson Registry.

**Patients and Methods:**

We analysed data from 162 WD patients with hepatic involvement and over one year of follow-up. Patients were classified as mild (no cirrhosis) or severe (with cirrhosis) at diagnosis. An “unstable pattern of transaminases” was defined as recurrent AST or ALT elevations. Unfavourable outcomes included new cirrhosis, elastography progression > 2 Kpa, liver transplant, or liver-related deaths. Logistic regression models were used to evaluate the impact of various factors on disease outcome.

**Results:**

Of 162 patients, 81.5% had mild disease at diagnosis. Most received chelators as first-line therapy, achieving an 81.4% one-year biochemical response. After a median follow-up of 17 years, 59% exhibited an unstable transaminase pattern, and 29% had an unfavourable outcome. Key factors associated with poor outcome included older age at diagnosis (OR = 1.03), lack of early biochemical response (OR = 0.19), advanced disease markers (platelet count, albumin), and an unstable transaminase pattern (OR = 2.92). Transaminase levels did not predict outcomes based on initial disease severity. Even patients with mild disease at diagnosis and persistently normal transaminases could experience progression over time, underscoring the need for more thorough follow-up evaluations.

**Conclusion:**

While transaminases are valuable for monitoring WD, they should be used alongside other biomarkers to better predict disease progression.

**Supplementary Information:**

The online version contains supplementary material available at 10.1186/s13023-025-03821-1.

## Background

Wilson disease (WD) is a rare hereditary metabolic disorder with variable clinical presentations, including hepatic, neurological, psychiatric, and ophthalmologic features, often in combination [1, 2]. Biallelic pathogenic mutations in the *ATP7B* gene result in defective biliary excretion of the excessive copper, and its accumulation in hepatocytes and other tissues [3, 4]. Early diagnosis and effective treatment are crucial to prevent progressive damage. In this regard, the latest guidelines recommend achieving a gradual reduction in liver enzymes to normal or near-normal levels as a target in patients with hepatic involvement, a goal typically reached within 6–12 months [1–3, 5].

After initial intense copper removal with chelating agents over the first year, the goal of maintenance therapy is to prevent copper re-accumulation in tissues while avoiding over-treatment [6, 7]. Parameters traditionally associated with this long-term stability include those related to copper homeostasis, specifically 24-h urinary copper excretion (UCE) levels [UCE < 75–100 µg in patients treated with zinc salts, or between 150 and 500 µg/day in patients on chelation therapy (trientine or penicillamine)], or bioavailable copper (traditionally named as “*free copper*”, and representing the non-ceruloplasmin-bound copper fraction or NCC levels below 50–75 µg/L in all patients. In the last years, the use of the classical NCC estimation obtained from the indirect calculation (NCC = total copper [µg/dL]—3.15 × ceruloplasmin [mg/dL]) has been progressively abandoned, due to its considerable variability, inaccuracy and uninterpretable results in up to 25% of patients [8]. New assays for NCC direct calculations have recently been developed [9] overcoming these limitations but unfortunately are not widely available in common clinical practice.

Additionally, transaminase levels are expected to remain within normal or near-normal ranges over time while on treatment, with isolated elevations of liver enzymes observed not to be predictive of hepatic dysfunction or deterioration [1, 2, 10]. Unfortunately, despite these targets being established in various guidelines, there is no single surrogate parameter that can reliably confirm clinical stability or predict the absence of long-term hepatic complications.

Furthermore, real-world experience shows considerable heterogeneity in the management and outcomes of therapy across centers, and even within the same center [11–13]. In a multicenter Spanish study, liver progression to de novo cirrhosis or decompensation was shown to reach 20% [11]. It´s been clearly established that prognosis in WD depends on early diagnosis and proper adherence to chronic treatment. Despite known limitations, copper homeostasis parameters help us determine stability in WD from a metabolic perspective, yet they do not always correlate with liver enzymes nor reflect the coexistence of additional hepatic comorbidities [1, 2, 14–16]. Our hypothesis was that patients with elevated transaminase levels during follow-up, compared to those with persistently normal levels, would have a poorer prognosis in terms of clinical liver outcomes. The primary objective was to define the predictive value of transaminase levels in Wilson disease.

## Methods

### Patients

The National Spanish Registry on Wilson Disease (AEEH Wilson-Registry) was started in 2021 supported by the Spanish Association for the Study of the Liver (AEEH, from their words in Spanish) with the aim of capturing WD natural history, clinical data and main treatment characteristics of patients throughout the country (www.aeeh.es/registro/registro-de-enfermedad-de-wilson/).

This multicenter study included all adult patients (≥ 18 years) diagnosed with WD (defined as Leipzig score > 3) with a minimum follow up since diagnosis of one year and included in the AEEH WD Registry up to November 2023. The protocol had been reviewed and approved by the ethical committee from the coordinating center (Hospital Clínic Barcelona, CEIM HCB/2021/1099) and approved afterwards by all Ethical committees in the participating centers throughout the country. All patients included in the Registry consented to the use of their clinical data for investigational purposes. Exclusion criteria were follow-up less than one year, liver transplant within one year after diagnosis, and patients classified as “pure neurological phenotypes” regardless of liver enzymes or potential underlying liver disease.

Variables that were collected included demographics (age and sex at diagnosis), presentation type [hepatic (either as acute or chronic liver abnormalities) or mixed (hepatic associated with another condition, usually neurological)], concomitant neurological involvement at diagnosis/pre-treatment, severity of initial hepatic involvement (fibrosis stage if available, presence of cirrhosis and/ or liver decompensation), type of initial therapy (D-Penicillamine/Trientine vs. Zinc vs. combinations), and change of therapy during follow up with the date and reason (adverse event/treatment failure/change to maintenance therapy/others). Of note, in the registry, no specific criteria were required to define cirrhosis, and it was the decision of the investigators in each center to check the box “cirrhosis”. However, this registry was developed by and for hepatologists in Spain, and thus the identification of cirrhosis according to the standard criteria (clinical, radiological and/ or biochemically) is expected to be accurate.

Patients were stratified in two groups according to hepatic severity at diagnosis or baseline: mild group 1 (without cirrhosis) vs. severe group 2 (with cirrhosis). Patients in group 1 were selected based on their baseline biopsy (fibrosis Metavir score 0 to 3). In case a baseline liver biopsy had not been done, transient elastography was used with a cut-off of ≤ 9.9 kpas. This cut-off was chosen based on the only large study correlating fibrosis stage measured through histological examination and elastography in recently diagnosed patients [17]. Patients with cirrhosis by either clinical evaluation (cirrhosis box checked), histology (Metavir score F4) or elastography (> 9.9 kpas) were included in group 2.

### Variables, time and outcome definitions

The Study Measure was liver function tests [AST, ALT, GGT (value and multiples of the upper limit of normal-ULN), total bilirubin, platelets, albumin]. The upper limit of normal for both transaminases (AST, ALT) was defined as 40 IU/L. Laboratory data was collected initially (at diagnosis or baseline = T0), and whenever available during further follow-up: at 1, 3, 5- and/or 10-years post-treatment initiation (defined as T1, T3, T5 and T10 respectively). Due to the retrospective nature of the registry, not all patients had available data at all these points. In addition, physicians were asked to collect whether patients had normalized liver enzymes with treatment (biochemical response within one year), and whether once normalized, liver transaminases had remained within normal range during follow-up or not (transaminase pattern overtime). In case of a lack of initial normalization and/or re-elevation once normalized, potential reasons were checked for. Additionally, in case of elevated transaminases during follow-up, it was determined whether the elevation was isolated (occurring on a single occasion) or persistent.

Based on this information, the “initial biochemical response” was defined as normalization of liver enzymes during the first year. The “pattern of transaminases” during follow-up collected data from subsequent visits was considered stable when liver transaminases remained always normal (or with an isolated elevation) vs unstable in those with persistent elevated transaminases and/or fluctuating levels between normal/elevated more than once.

The patient’s outcome was based on clinical and elastography results at 10 years or at their last available follow-up. “Favorable Outcome” was defined by the absence of clinical events defining liver decompensation (ascites, variceal bleeding, encephalopathy, acute-on-chronic liver failure-ACLF), no development of *the novo* liver cancer, lack of neurologic progression together with histologic and/or elastography stability or improvement. In contrast, “Unfavorable Outcome “was defined as progression to cirrhosis (in those with no cirrhosis at baseline), clinical decompensation, including the need for transplantation or death from hepatic causes, development of de novo liver cancer, or de novo neurologic manifestations and/or worsening of prior neuropsychiatric problems. In addition, in those without baseline cirrhosis, fibrosis progression (defined by increase in at least 1 unit of fibrosis in histology or > 2 Kpas in elastography) was also considered as “unfavorable outcome”. For those with baseline cirrhosis, absence of elastography improvement with persistent results above the 9.9 kpas threshold was also considered as a poor outcome measure.

### Statistical analysis

The descriptive analysis provides the most relevant statistics for all variables collected in the research, mean, standard deviation, minimum, maximum, median, and 25th and 75th percentiles (for continuous parameters) as well as absolute and relative frequencies (for categorical parameters). The inferential analysis aims to study the association between these variables and the outcome in each group (mild and severe group) separately and jointly. Simple binary logistic regression models were estimated to explain the probability of unfavourable progression over the follow-up period based on the independent variables. The odds ratio and 95% confidence intervals for the unadjusted association are provided. Independent variables identified as significant (*p* < 0.05) or relevant (*p* < 0.01) are used for the dual estimation of a multiple model and subsequent adjusted odds ratios. Most specifically, the pattern of transaminases during follow-up was analysed as a predictor of outcome in each group separately and jointly. The significance level used in the analyses was 5% (α = 0.05).

## Results

### Study group/Baseline characteristics

Of a total of 228 patients with WD included in the AEEH WD registry with a Leipzig score > 3 as of November 2023, 66 were excluded due to (i) follow-up less than one year (n = 29), liver transplant within one year after diagnosis (n = 4), and pure neurological phenotypes (n = 33). The final study group consisted of 162 patients with hepatic or mixed WD phenotype with more than one year of follow-up since diagnosis.

**Fig. 1:**
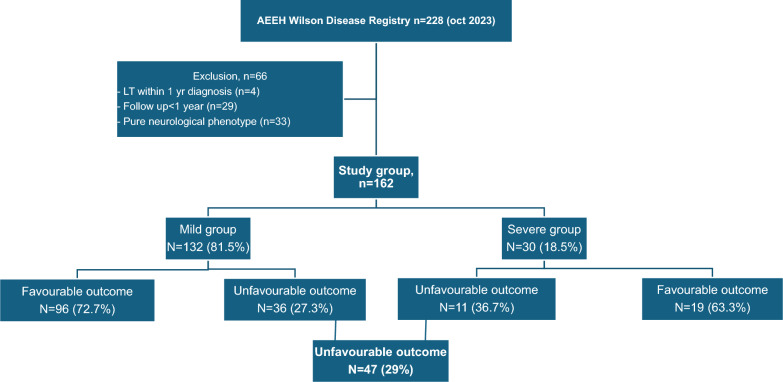
Patient disposition. *Mild group* defined as patients without cirrhosis or clinical decompensation at baseline. *Severe group* defined as patients with cirrhosis with/without decompensation at baseline. *Stable transaminase pattern* defined as normal liver enzymes during follow up once biochemical response was reached with first line therapy. *Unstable transaminase pattern* defined as altered liver enzymes during follow up after first line therapy (persistent elevated transaminases and/or fluctuating levels between normal/elevated)

Most patients were classified as mild (n = 132, 81.5%) (Table 1 and Fig. 1). The median age at diagnosis was 12 years (IQR 25–75%: 8–22), and significantly lower in the mild onset group (11.5 vs. 16.5 years; *p* = 0.018). Males accounted for 55.5% with similar sex distribution across the two groups. The median Leipzig score was 7 (5–8). Most presented with chronic hepatitis, with only 13% presenting with acute forms. Only a few had mild neuropsychiatric symptoms reported at diagnosis (4.5%).

**Table 1:** Baseline features of the patient population (at diagnosis) *

Variables	All (n = 162)	Mild, n = 132	Severe, n = 30	*P* value
Sex (% men)	89 (55.3%)	74 (56.5%)	15 (50%)	0.519
Duration of FU (yrs)	17 (11.1–25.1)	18.8 (12.3–25.2)	14.9 (7.6–20.3)	0.451
Age at diagnosis (yrs)	12 (8–22)	11.5 (8–20)	16.5 (12–34)	0.018
Leipzig score	7 (5–8)	7 (5–8)	8 (6–9)	0.088
Acute hepatitis as clinical presentation (%)	21 (13%)	15 (11.4%)	6 (20%)	0.204
Concomitant neuropsychiatric manifestations (%)	7 (4.5%)	6 (4.8%)	1 (3.4%)	0.759
Liver biopsy at diagnosis (%) *	121 (74.7%)	98 (74.3%)	23 (76.7%)	0.216
Fibrosis staging at Liver Biopsy (METAVIR) F0-2 F3 F4	69 (68.4%) 11 (10.9%) 21 (20.8%)	68 (86%) 11 (14%) 0	1 (4.5%) 0 21 (95.5%)	< 0.001
Steatosis at LB (%)	83/104 (80%)	74/87 (85%)	9/17 (53%)	0.003
Elastography at baseline Median (25-75th) (Kpas)	10 (6.2%) 6.4 (5.5–21.3)	6 (4.5%) 5.9 (4.4–6.4)	4 (13.3%) 25.7 (21.3–32.8)	0.229 0.024
First line therapy (%) Chelating therapy Salt zinc therapy Combined therapy	114 (70.4%) 38 (23.5%) 9 (5.5%)	93 (70.5%) 37 (28%) 2 (1.5%)	21 (70%) 1 (3.3%) 7 (23.3%)	0.961 0.004 < 0.001
AST (IU/L) at T0 Times ULN	81.5 (42–112) 2.04 (1.05–2.80)	82 (42.5–116.5) 2.05 (1.06–2.91)	73.5 (32–111) 1.84 (0.8–2.78)	0.478 0.478
ALT (IU/L) at T0 Times ULN	139 (65–229) 3.48 (1.63–5.7)	170 (84–251) 4.25 (2.1–6.3)	65 (26–139) 1.63 (0.65–3.48)	0.002 0.002
% with elevated AST ALT	91/114 (79.8%) 97/115 (84.3%)	72/88 (81.8%) 80/89 (89.9%)	19/26 (73.1%) 17/26 (65.4%)	0.329 0.002
GGT (IU/L) at T0	63 (42–104)	61 (40–84)	99 (43–148)	0.105
Platelet count (/mm3) at T0	253.5 (208–316)	255 (220–324)	211 (181–253)	0.041
Albumin (g/L) at T0	43 (38–44)	44 (43–46)	34 (26–40)	< 0.001
Bilirubin (mg/dl) at T0	0.7 (0.5–1)	0.6 (0.4–0.9)	1.5 (0.8–2.2)	0.004
Ceruloplasmin (g/L) at T0	5 (3–8.5)	4 (3–7)	6 (4–12)	0.450
Cupruria (mcg/24 h) at T0	149.5 (48–400)	97 (33–255)	400 (218–624)	0.016

Denominators differ based on available data for each variable

^*^not all patients with a biopsy done at baseline had information on liver fibrosis

Many patients lacked baseline elastography data due to historical diagnosis, but 121 (74.7%) had a baseline biopsy available with no differences between groups; the majority showed mild (51.5%) or moderate (26.5%) inflammatory activity, and 80% had some degree of hepatic steatosis. Among the 101 patients with recorded Metavir stage at diagnosis, 26 had F0 (25.7%), 19 F1 (18.8%), 24 F2 (23.8%), 11 F3 (10.9%), and 21 F4 (20.8%). Per definition, all F4 patients were included in the severe onset group. One F1 staged patient was also included in the severe group, due to concomitant imaging study showing indirect signs of cirrhosis (as per the center investigator). Baseline stiffness, available for only 10 patients, was 6.4 kPa (5.5–21.3), significantly lower in the mild group [5.9 (4.4–6.4) versus 25.7 (21.3–32.8), *p* = 0.024]. Three patients were included in group 2 (severe) for elastography reasons.

Considering biopsy, elastography, or clinical-imaging-analytical data, 30 patients had cirrhosis at diagnosis and composed the severe group 2 (21.1%) (Table 1). Of these, 12 had already presented with some decompensation, mainly ascites (n = 12), with two diagnosed in the context of acute on chronic liver failure (ACLF).

### First-line treatment and biochemical initial response to therapy

Regarding initial treatment, most patients received chelation therapy (70.4%), with a similar proportion in both groups. Monotherapy with zinc salts was more common in the mild group (28%) than in the severe group (3.3%), while combination therapy was more frequent in the severe group (23.3% vs. 1.5% in the mild group) (Table 1).

The evolution of laboratory parameters is shown in Suppl Table 1. Overall, there was a gradual reduction in transaminases and GGT, with a minor rebound at the 10-year mark, with no differences between groups.

One hundred and fourteen out of 140 with available information (81.4%) normalized transaminases within 1 year of therapy. The rate of biochemical response was similar across the two groups, 82% in the mild group (98/119) and 76% in the severe (16/21) (*p* = 0.503). Stability in other laboratory parameters, including total bilirubin, albumin, and platelets, was observed without significant differences between groups (Suppl Table 1).

### Transaminases pattern overtime: stable / unstable

The median follow-up time since diagnosis was 17 years (11.1–25.1), close to 19 (12–25) in the mild group and 15 (7.5–20) in the severe group. Most patients (143, 88.3%) had at least one AST and/or ALT measurement available during follow-up yet only 47 (29%) had data in all the defined time-points. According to our established definitions, most patients had an “unstable transaminase pattern” (n = 82, 59%) over time, with slightly higher frequency in those with severe baseline presentations (severe, n = 14/21; 66.7% vs. mild, n = 68/118; 57.6%; *p* = 0.438) (Table 2).

**Table 2:** Transaminase pattern over time based on baseline disease severity*

	Mild group (n = 118)	Severe group (n = 21)
Stable (n = 57)	50 (42.4%)	7 (33.3%)
Unstable (n = 82)	68 (57.6%)	14 (66.7%)

^*^Data not available for all patients

Mild group defined as patients without cirrhosis or clinical decompensation at baseline

Severe group defined as patients with cirrhosis with/without decompensation at baseline

Stable transaminase pattern defined as normal liver enzymes during follow up once biochemical response was reached with first line therapy

Unstable transaminase pattern defined as altered liver enzymes during follow up after first line therapy (persistent elevated transaminases and/or fluctuating levels between normal/elevated)

### Unfavourable outcomes and predictive variables of outcomes (including baseline characteristics, initial biochemical response and transaminase pattern)

A total of 47 patients (29%) experienced “Unfavourable Outcome”: 27.3% in the mild group (n = 36/132) and 36.7% in the severe group (n = 11/30) (Fig. 1). For the mild group, the unfavourable outcomes reported in 36 patients were stiffness progression > 2 Kpas over time (n = 19), cirrhosis development (n = 15), liver decompensation (n = 8), LT (n = 4) and liver related deaths (n = 3). Of note, each patient could reach several endpoints defining unfavourable evolution. In the severe group, 11 patients had unfavourable outcomes due to cirrhosis without elastography improvement (n = 11), decompensation (n = 6), liver cancer (n = 1), LT (n = 2), and liver related death (n = 1).

The results of the binary logistic regression analysis with the unadjusted odds for unfavourable outcome are shown in Table 3**.** In essence, older patients at diagnosis had a worse outcome (OR = 1.03; *p* = 0.010); each additional year of delay added 3% risk of unfavourable outcome. The presence of baseline steatosis at liver biopsy was protective such that unfavourable outcome occurred in 26.5% (n = 22/83) of those with baseline steatosis, as opposed to 57.1% (n = 12/21) in those without (OR = 0.27; *p* = 0.010). Yet, this variable was only available in 104 patients. Baseline platelet count was the strongest factor associated with clinical outcomes, with lower platelet count in those with unfavourable outcome (216,500 vs 260,500/mm^3^; OR = 0.985; *p* = 0.008). In addition, at T1, lower albumin levels were associated with unfavourable outcome (OR = 0.782; *p* = 0.016) at long-term, yet this variable was only available in 50 patients. Finally, initial biochemical response within one year of therapy was predictive of clinical outcomes, reducing the risk of unfavourable events by 81%. In those with liver transaminase normalization, an unfavourable outcome was only reached by 20.2% vs 57.7% in those who did not normalize liver enzymes at their T1 evaluation (OR = 0.19; *p* < 0.001). Finally, the pattern of transaminases following initial response was also statistically associated with outcome, such that an unfavourable outcome was reached by 15.8% of those with stable pattern vs 35.45% of those with unstable pattern (OR = 2.92; *p* = 0.013).

**Table 3:** Association between predictive variables and outcome

	MILD GROUP (n = 132)				SEVERE GROUP (n = 30)				TOTAL (n = 162)			
Outcomes	Total (n*)	Favorable	Unfavorable	*p*-value	Total	Favorable	Unfavorable	p-value		Favorable	Unfavorable	*p*-value
Age (yrs)	11.5 (8–20)	10 (8–15)	18 (9.5–28)	**OR = 1.03 *p* = 0.025**	16.5 (12–34)	11.5 (9–34)	27 (15–31)	0.321	12 (8–22)	9 (8–17)	19 (10–30)	**OR = 1.03 *p* = 0.010**
Men (%)	74	53 (71.5%)	21 (28.5%)	0.625	15	9 (60%)	6 (40%)	0.705	89	62 (70%)	27 (30%)	0.582
Leipzig score	7 (5–8)	7 (5–8)	6 (4–8)	0.287	8 (6–9)	8 (6–9)	7 (5–9)	0.306	7 (5–8)	7 (6–8)	6 (4–8)	0.213
Acute hepatitis phenotype (%)	15	9 (60%)	6 (40%)	0.246	6	3 (50%)	3 (50%)	0.453	21	12 (57%)	9 (43%)	0.139
Neurologic manifestations (%)	6	6 (100%)	0	–	1	0	1 (100%)	–	7	6 (86%)	1 (14%)	0.429
Steatosis at diagnosis (%)	74	56 (75.7%)	18 (24.3%)	**OR = 0.20 *p* = 0.011**	9	5 (55.5%)	4 (44.5%)	0.819	83	61 (73.5%)	22 (26.5%)	**OR = 0.27 *p* = 0.010**
Liver Stiffness at baseline (Kpa)	5.8 (3–8.3)	6.2 (5.8–8.3)	5.3 (3–8.3)	**OR = 0.84 *p* = 0.054**	15 (15–15)	15 (15–15)	15 (15–15)	0.475	8.3 (5–11)	8.3 (5.8–11)	7 (3–13)	0.846
Chelator at baseline (%)	95	67 (70%)	28 (30%)	0.365	28	18 (64%)	10 (36%)	–	123	85 (69.1%)	38 (30.9%)	0.244
Adverse events (%)	41	30 (73.2%)	11 (26.8%)	0.939	13	9 (69%)	4 (31%)	0.559	54	39 (72%)	15 (28%)	0.807
Suboptimal adherence to Treatment (%)	17	13 (76.5%)	4 (23.5	0.959	4	3 (75%)	1 (25%)	0.662	9	5 (56%)	4 (44%)	0.903
Follow Up time (yrs)	18.8 (12.3–25.2)	17.8 (11.2–24)	19.8 (13.5–26.3)	0.284	14.9 (7.6–20.3)	16.4 (8.9–20)	10.4 (3.7–37.5)	0.596	17 (11–25.1)	18.8 (12.3–25.2)	14.9 (7.6–20.3)	0.269
AST T0 (IU/L)	82 (42–116)	84 (49–117)	75 (36–105)	0.403	73.5 (32–111)	97 (29–112)	56 (32–104)	0.572	81.5 (42–112)	85 (49–116)	65 (36–104)	0.275
ALT T0 (IU/L)	170 (84–251)	178 (84–293)	152.5 (72–200)	0.344	65 (26–139)	65 (29–161)	65 (21–139)	0.344	139 (65–229)	157 (70–251)	114 (54–182)	0.101
AST T0 elevated (%) ALT T0 elevated (%)	72 80	56 (78%) 61 (76%)	16 (22%) 19 (24%)	0.208 0.530	19 17	11 (58%) 11 (65%)	8 (42%) 6 (35%)	0.973 0.324	91 97	67 (73.5%) 72 (74.2%)	24 (26.5%) 25 (25.8%)	0.232 0.434
GGT T0 (IU/L)	61 (40–84)	57.5 (40–80)	66 (40–87)	0.262	99 (43–148)	108 (45–215)	94(27–130)	0.360	63 (42–104)	60 (43–103)	67 (37–106)	0.441
Total bilirubin T0 (mg/dL)	0.6 (0.4–0.9)	0.6 (0.4–0.9)	0.7 (0.6–1)	**OR = 3.83 *p* = 0.041**	1.5 (0.8–2.2)	1.5 (0.8–2.2)	1.4 (0.8–2.5)	0.649	0.7 (0.5–1)	0.6 (0.4–0.9)	0.9 (0.6–1.5)	0.357
Albumin T0 (g/L)	44 (43–46)	44 (43–46)	44 (43–44)	0.490	34 (26–40)	31.5(26–40)	37 (28–39)	0.721	43 (38–44)	43 (40–46)	40 (36–43)	0.130
Platelet count T0 (/mm3)	255 (220–324)	266 (236–346)	221.5 (194–248)	**OR = 0.985 *p* = 0.015**	211 (181–253)	211 (181–253)	204 (180–253)	0.966	253.5 (208–316)	260.5 (222–327)	216.5 (192–248)	**OR = 0.985 *p* = 0.008**
Urinary copper T0 (ug/24 h)	97 (33–255)	105 (36–245)	68 (23–330)	0.595	400(218–624)	412.5 (285–606)	276 (107–850)	0.482	149 (48–400)	149 (47–412)	204 (51–390)	0.450
Free Copper T0 (ug/dl)	10 (7–15)	11 (6–16)	10 (8–14)	0.325	14.4 (7.5–37)	15.8 (8–40)	13 (6–34)	0.530	11 (7–19)	11 (8–20)	10 (7–14)	0.392
Ceruplasmin T0 (mg/dl)	4 (3–7)	4 (3–7)	3 (2–9)	0.630	6 (4–12)	5 (3–8)	7 (4–14.5)	0.150	4.9 (3–8.5)	4.9 (3–7)	5 (3–11)	0.768
AST T1 elevated (%) ALT T1 elevated (%)	26 40	19 (73%) 30 (75%)	7 (27%) 10 (25%)	0.282 0.237	9 10	7 (78%) 7 (70%)	2 (22%) 3 (30%)	0.325 0.738	35 50	24 (68.6%) 37 (74%)	11 (31.4%) 13 (26%)	0.131 0.450
Biochemical response to first treatment (%) Yes No	98 21	80 (81.6%) 10 (47.6%)	18(18.4%) 11(52.4%)	**OR = 4.89 *p* = 0.002**	16 5	11 (68.8%) 1 (20%)	5 (31.3%) 4 (80%)	**OR = 8.80 *p* = 0.080**	114 26	91 (79.8%) 11 (42.3%)	23 (20.2%) 15 (57.7%)	**OR = 5.40 *p* < 0.001**
Transaminase pattern over time (%) Stable Unstable	50 68	42 (84%) 47 (69.1%)	8 (16%) 21 (30.9%)	**OR = 2.35 *p* = 0.068**	7 14	6 (85.7%) 6 (42.9%)	1 (14.3%) 8 (57.1%)	**OR = 8.00 *p* = 0.085**	57 82	48 (84.2%) 53 (64.6%)	9 (15.8%) 29 (35.4%)	**OR = 2.92 *p* = 0.013**

A multivariate analysis was performed including variables with a p value < 0.1 but excluding those with significant missing values (steatosis, biochemical data at T1). Low platelets count and unstable transaminase pattern were still predictive of unfavourable outcome. The unstable pattern (i.e. persistent elevated enzymes and/or re-elevation following initial normalization) multiplied by 10 the risk of poor outcome at long-term (Table 4).

**Table 4:** Independent factors associated with unfavourable outcome

	Category	OR	95% CI	*P* value
OVERALL COHORT				
Biochemical response to first line therapy	Yes No	1 3.13	0.69–14.29	0.139
Transaminase pattern	Stable Unstable	1 9.96	1.69–58.6	0.011*
Age		1.01	0.96–1.07	0.706
Platelet count T0		0.989	0.981–0.996	0.004**
GROUP 1 (MILD DISEASE) (n = 132)	Category	OR	95% CI	P value
Biochemical response to first line therapy	Yes No	1 9.09	1.92–100	0.032*
Age		0.96	0.89–1.05	0.368
Platelet count T0		0.979	0.960–0.998	0.027*
Stiffness T0		0.870	0.602–1.258	0.459
GROUP 2 (SEVERE) (n = 30)	Category	OR	95% CI	P value
Biochemical response to first line therapy	Yes No	1 5.00	0.39–50	0.217
Biochemical pattern	Stable Unstable	1 4.80	0.40–58	0.217

^*^p < 0,05; **p < 0,01; ***p < 0,001

We then did similar analyses by stratifying patients according to their baseline disease (mild vs severe). In multivariable analysis in group 1 (mild disease), low platelet counts at baseline (OR = 0.979; *p* = 0.027), and lack of normalization of liver enzymes with therapy (initial biochemical response) (OR = 0.11; *p* = 0.032) predicted unfavourable outcome. The pattern of transaminases over time did not predict outcome. In group 2, none of the variables could explain independently the outcome.

Table 5 shows the specific associations between initial biochemical response to therapy (within 1 year) as well as transaminase pattern overtime and clinical outcome. As specified before, initial biochemical treatment response was significantly predictive of favourable outcome in the MILD group whereas a stable transaminase pattern independently predicted favourable outcome in the overall population (Table 4), yet none of these conditions were fully predictive of outcome. For instance, 15.8% of patients with stable transaminase pattern over time, as well as 18.4% of patients with mild disease at baseline who normalized liver enzymes within one year of therapy, had unfavourable clinical outcomes. In turn, the lack of biochemical response as well as unstable transaminase pattern did not systematically predict unfavourable outcome. Of note, 47.6% of patients with mild disease at baseline who did not normalize liver enzymes with first line therapy had a favourable outcome whereas 64.6% of patients with unstable transaminase pattern eventually attained a favourable outcome (Table 5).

**Table 5:** Association between initial biochemical response to therapy (Table 5 A) and subsequent transaminase pattern (Table 5 B) and outcome

	Group 1 (Mild Baseline Disease)				Group 2 (Severe Baseline Disease)				Whole Study Cohort			
		Favorable	Unfavourable	OR *p*-value		Favorable	Unfavourable	OR *p*-value		Favorable	Unfavourable	OR *p*-value
	(A) Initial biochemical response and outcome											
	n	N (%)	N (%)		n	N (%)	N (%)		n	N (%)	N (%)	
Total	119	90 (75.6%)	29 (24.4%)		21	12 (57.1%)	9 (42.9%)		140	102 (72.9%)	38 (27.1%)	
YES	98	80 (81.6%)	18 (18.4%)	1	16	11 (68.8%)	5 (31.3%)	1	114	91 (79.8%)	23 (20.2%)	1
NO	21	10 (47.6%)	11 (52.4%)	4.89 *p* = 0.002	5	1 (20%)	4 (80%)	8.00 *p *= 0.080	26	11 (42.3%)	15 (57.7%)	5.40 *p* < 0.001
	(B) Transaminase pattern and outcome											
Total	118	89 (75.4%)	29 (24.6%)		21	12 (57.1%)	9 (42.9%)		139	101 (72.7%)	38 (27.3%)	
Stable	50	42 (84%)	8 (16%)	1	7	6 (85.7%)	1 (14.3%)	1	57	48 (84.2%)	9 (15.8%)	1
Unstable	68	47 (69.1%)	21 (30.9%)	2.35 *p* = 0.068	14	6 (42.9%)	8 (57.1%)	8.00 *p* = 0.085	82	53 (64.6%)	29 (35.4%)	2.92 *p* = 0.013

## Discussion

Assessment of Wilson Disease patients in the long term is plagued with challenges due to the lack of good reliable markers defining clinical stability with lack of disease progression. In patients with hepatic presentation, normalization or near normalization of liver enzymes (to < 1.5 ULN) is considered the goal of therapy. Despite therapy though, a proportion of patients with WD will progress to cirrhosis and premature death. This might be due to multiple reasons, such as adherence issues, suboptimal therapy or monitoring, late diagnosis or the presence of hepatic comorbidities, among others [1–3, 5, 6, 18–20]. The alcohol and metabolic-associated liver disease pandemics are likely affecting WD patients as life expectancy is prolonged, with otherwise well controlled copper metabolism. To what extent may the liver transaminase pattern/ abnormalities capture these circumstances and may justify unfavorable outcomes in the long-term is still unknown. In one small study on 12 WD patients treated with zinc and/or penicillamine who underwent multiple follow up liver biopsies, there was no association between the histological findings and serum transaminases [13]. In another study, 35% of children with WD-related liver disease had persistent hypertransaminasemia despite treatment with penicillamine or zinc. Interestingly, despite longstanding abnormalities, no patient showed worsening of liver disease or developed other WD-related symptoms after a follow-up of 53 months [12]. Finally, in a recent multisite registry, 64% of patients with an average of > 10 yrs of medical treatment had abnormal ALT or AST [10].

In this study we aimed at interrogating our data from the Spanish Wilson AEEH Registry and explore the potential impact of unstable transaminase pattern on predicting unfavorable hepatic outcomes. Our hypothesis assumed these abnormal transaminases would be reflective of any liver damage, either due to uncontrolled WD and/or the presence of comorbidities, with potential detrimental effects for patients in the long-term.

We determined the predictive value of transaminases in WD both in patients with initial advanced liver disease as well as in those with mild disease. Of note, determining advanced disease in WD is a first challenge given the lack of association between liver enzymes and the degree of underlying liver injury as already reported for other liver diseases [21]. Cirrhosis can be diagnosed through a liver biopsy and/or radiological means [22]. Unfortunately, studies assessing the role of elastography in diagnosing advanced liver disease in WD are still limited with inconsistent results [17, 23]. Furthermore, results might differ whenever naive or treated WD patients are considered. The effect of copper chelating therapy on reducing elastographic stiffness in WD independently of liver fibrosis has already been suggested [17, 24]. Although limited data is available, this reduction over time would potentially mimic what is seen in other liver diseases after removal of the primary aetiological factor [25]. Whether this effect is due to a reduction of the inflammatory component, influenced by copper storage or even due to fibrosis reversal is still unknown. In our study, due to its retrospective nature, data at diagnosis was mostly obtained from liver biopsy, which was the basis to diagnose the presence of cirrhosis and the current gold standard in WD. In a few instances though, elastography was used to diagnose cirrhosis at baseline with a cut-off of 9.9 kpas based on the largest study showing an association between liver histology with elastographic findings among naïve WD patients [17]. Whether the same is true for cirrhosis identification in the long-term among treated patients is yet to be established, but the observation of significant increasing stiffness over time (as defined in our elastographic unfavorable outcome of 2 kPa increase) could be read as a negative factor. In fact, in the previous referred study by Paternostro et al., only 5.9% of non-cirrhotic WD patients showed “progression” to cirrhotic LSM values, while 30.8% of cirrhotic WD patients showed LSM values suggestive of cirrhosis “regression” during a median follow-up of 46 months [17].

Of note, while we acknowledge that ALT values may vary based on sex and BMI, and lower thresholds have been proposed in recent studies [21], we decided to use the same cut-off for both AST and ALT regardless of BMI and gender because we were dealing with a retrospective study spanning many years, and multiple centers with different laboratories implicated, and some historical data (such as BMI) were not available at medical chart review in many cases.

The main findings from this study can be summarized as follows: (i) as already reported in the literature [1–3, 7], most patients normalize liver enzymes with therapy; (ii) a significant proportion of patients, regardless of baseline disease severity, show a re-elevation of liver enzymes in the long-term. Ngwanou and colleagues [26] reported similar trends in transaminases among a longitudinal cohort of WD children, which was attributed to problems in adherence. Liver tests abnormalities indeed can be associated with lack of adherence, as well as suboptimal therapy (either dose or drug choice), or presence of comorbidities, but this evaluation is beyond the scope and capacity of this registry. Yet the registry allows for free text to discuss potential reasons for re-elevation of liver enzymes, and in most cases the two main reasons reported were lack of compliance and concomitant steatotic liver disease. Only in 13/56 cases where an explanation was provided, there was no apparent reason for the unstable pattern of liver enzymes (data not shown); (iii) while achieving a biochemical response and maintaining normal liver enzymes is frequently associated with good prognosis, a stable enzyme pattern does not guarantee this favorable outcome; yet based on our series, this is a small percentage of patients; (iv) alternatively, despite persistent transaminase elevation, a significant proportion of patients, including those with advanced liver disease at baseline, have a favorable outcome after a follow up close to 20 years; (v) low platelet count, indirect marker of portal hypertension, predicts unfavorable prognosis.

The lack of association between initial treatment response to first line therapy and long-term outcome in the overall cohort can be explained by the long natural history of WD patients where treatments are frequently modified, and comorbidities may develop during follow-up. Indeed, patients had a median follow-up of 17 years and therapy switches were frequent over time. In addition, transaminases were only recorded at large intervals (1, 3, 5 and 10 years) which may have biased these results. Moreover, patients were not homogeneously evaluated, as the registry included data from more than 40 participating centers in Spain. Of note, initial biochemical treatment response was significantly predictive of favourable outcome in the group of patients with baseline mild disease, the largest group in our study, similar to recent observations reported in autoimmune hepatitis [27, 28]. As in AIH, the absence of such a response might be used to identify patients that might benefit from intensified monitoring and escalation of treatment.

Furthermore, many paediatricians strive for "normal" values being well below the upper limit of normal. The benefit for long-term transplant-free survival of having very low normal aminotransferases (eg, < 0.5 × ULN) is unclear. Unfortunately, the number of patients in our study with liver enzymes < 0.5 × ULN was too small (n = 3) to make meaningful analyses.

Other limitations that need to be highlighted include the small sample size, particularly those with severe baseline liver disease; the lack of adequate information on comorbidities (alcohol consumption, weight, or diabetes among others) and the lack of proper data on treatment adherence. These limitations apply to all observational registries and reflect the complexity of WD treatment and monitoring.

Despite these drawbacks, our results remain relevant as they reflect current clinical practice and disease progression in Spain from a hepatological perspective on WD. While our study was unable to demonstrate an association between abnormal transaminase patterns and unfavourable outcomes, this should not be interpreted as a lack of association. In fact, most patients had positive long-term outcomes, regardless of baseline disease severity and despite abnormal liver test results, as previously reported in larger European cohorts [29]. An alternative interpretation would necessitate a broader discussion on how to optimize the management of mild WD cases that progressed over time in our practice. Additionally, it serves as a cautionary note for physicians: liver transaminases are clinically relevant but should never be assessed in isolation, as they neither capture the full spectrum of WD-related damage nor guarantee a favourable prognosis. This principle may also apply to neuro-Wilson patients, although they were deliberately excluded from this analysis to minimize variability.

Monitoring Wilson’s disease is inherently complex, requiring the integration of copper biomarkers, routine liver function tests, treatment adherence assessments, and clinical evaluations to ensure optimal patient outcomes. Elevated transaminase levels may indicate inadequate treatment—necessitating dose adjustments, improved adherence, or even a therapeutic switch—but they can also result from coexisting conditions, particularly in WD patients transitioning into adulthood. Therefore, the detection of abnormal liver enzymes should always prompt a thorough evaluation. Conversely, the presence of normal liver enzyme levels should not lead to complacency, as other critical disease markers must also be assessed.

Unfortunately, current WD monitoring tools have significant limitations. Free copper calculation (derived from ceruloplasmin and total copper) is uninterpretable in approximately 25% of patients [30]. Urinary copper excretion (UCE), though useful, exhibits significant variability between visits due to fluctuations in dietary copper intake and the cupriuretic effects of chelators [6–8, 30]. Off-treatment UCE may help reduce this variability over time [2] and can aid in detecting non-adherence [31], but it has yet to become standard practice. Non-ceruloplasmin-bound copper (NCC) is considered the most reliable surrogate marker of copper status [32], but target ranges for follow-up still need to be clearly defined [16]. There is an urgent need to establish the most accurate and cost-effective biomarker for WD, and promising research is ongoing in this field. In the meantime, a combined approach using multiple available tools appears to be the best strategy.

In conclusion, we have assessed long-term hepatic outcomes in a large group of WD patients in Spain using the Wilson AEEH Registry. This collaborative effort has been instrumental in characterizing our patient population and identifying key limitations in current clinical practice. Notably, we have demonstrated that a significant proportion of patients experienced unfavourable long-term outcomes despite mild disease at diagnosis, underscoring the challenges in long-term patient monitoring. Abnormal liver enzyme patterns did not emerge as the primary negative predictive factor for disease progression, whereas an early biochemical response appeared to be crucial. However, the retrospective nature of data collection within the registry may have limited the depth of our analysis. We believe there is significant room for improvement, and clinicians should recognize the importance of combining liver enzyme assessments with biomarkers to ensure disease stability. Additionally, certain patients may benefit from specialized care at expert centers, where advanced diagnostic tools can help identify additional contributing factors.

## Supplementary Information

Supplementary file 1.

## Data Availability

Anonymized data will be made available on reasonable request. The Wilson Disease Registry belongs to the Spanish Liver Association (AEEH). More information is available at https://aeeh.es/wp-content/uploads/2024/09/CEIM_WilsonRegistroAEEH_V4.pdf

## Abbreviations

ACLF: Acute on chronic liver failure
AEEH: Asociación española para el estudio del hígado
ALT: Alanine aminotransferase
AST: Aspartate aminotransferase
F: Fibrosis
GGT: Gammaglutamyl transpeptidase
LT: Liver transplantation
NCC: Non-ceruloplasmin-bound copper fraction
OR: Odd ratio
T0: Time 0 or baseline
UCE: Urinary copper excretion
ULN: Upper limit of normal
WD: Wilson disease
