# Supplementary material for: Predictive value of liver enzymes in long-term prognosis of hepatic Wilson disease: results from the Wilson AEEH registry
Source: Orphanet J Rare Dis. 2025 Jun 7;20:288. doi: 10.1186/s13023-025-03821-1 (PMC12145598; doi:10.1186/s13023-025-03821-1)
Supplement: Supplementary file 1 [file 13023_2025_3821_MOESM1_ESM.docx]

**Suppl Table 1. Evolution of analytical & elastography parameters (median values)**.

|  | **MILD** | **Severe** | **TOTAL** |
| --- | --- | --- | --- |
| AST (UI/L)  T0  T1  T3  T5  T10 | 82 (42.5-116.5)  36 (28-48.5)  29 (22.5-38.5)  29 (22-37)  26 (20-40) | 73.5 (32-111)  40 (29-56)  27 (20.6-45)  23 (21-34)  32 (22-37) | 81.5 (42-112)  36 (28-49)  27 (22-39)  28 (22-37)  27 (21-27) |
| AST (multiple ULN)  T0  T1  T3  T5  T10 | 2.05 (1.06-2.91)  0.9 (0.7-1.21)  0.72 (0.56-0.96)  0.73 (0.55-0.93)  0.65 (0.5-1) | 1.84 (0.8-2.8)  1 (0.73-1.4)  0.7 (0.52-1.13)  0.58 (0.53-0.85)  0.8 (0.55-0.93) | 2.04 (1.05-2.80)  0.90 (0.70-1.23)  0.68 (0.55-0.98)  0.7 (0.55-0.93)  0.68 (0.53-0.93) |
| ALT (UI/L)  T0  T1  T3  T5  T10 | 170 (84-251)  47.5 (34-84)  29 823-58)  40 (25-66-)  36 (25-72) | 65 (26-139)  44 (30-61)  30 (19-61)  32 (22-51)  48.5 (30-105) | 139 (65-229)  47.5 (32-81)  29.5 (21-60)  38.7 (23-65.5)  41 (25-78) |
| ALT (multiple ULN)  T0  T1  T3  T5  T10 | 4.25 (2.10-6.28)  1.19 (0.85-2.1)  0.73 (0.58-1.45)  1 (0.63-1.65)  0.9 (0.63-1.8) | (0.65-3.48)  1.10 (0.75-1.53)  0.75 (0.48-1.53)  0.8 (0.55-1.28)  1.21 (0.75-2.63) | 3.48 (1.63-5.73)  1.19 (0.8-2.03)  0.74 (0.53-1.5)  0.97 (0.58-1.64)  1 (0.63-1.95) |
| GGT (UI/L)  T0  T1  T3  T5  T10 | 61 (40-84)  25 (18-43)  22 (13-33)  22 (15-40)  26 (15-38) | 99 (43-148)  28 (23-47)  20 (14-28)  20 (14-37)  26 (19-34) | 63 (42-104)  26 (18-43)  21 (13-33)  21.5 (15-38)  26 (16-38) |
| Total bilirubin (mg/dL)  T0  T1  T3  T5  T10 | 0.60 (0.4-0.9)  0.60 (0.5-0.8)  0.57 (0.4-0.8)  0.55 (0.36-0.76)  0.60 (0.5-0.81) | 1.5 (0.8-2.2)  0.7 (0.6-1)  0.9 (0.62-1.3)  0.7 (0.6-1.3)  1.0 (0.8-1.2) | 0.7 (0.5-1))  0.6 (0.5-0.9)  0.6 (0.45-0.9)  0.6 (0.4-0.82)  0.69 (0.5-0.93) |
| Albumin (g/L)  T0  T1  T3  T5  T10 | 44 (43-46)  44 (42-46)  45 (43-47)  45 (43-47)  45 (43-47) | 34 (26-40)  43 (40-47)  46.5 (44-47)  45 (44-46)  46 (42-48) | 43 (38-44)  44 (42-46)  46 (43-47)  45 (43-47)  45 (42-47) |
| Platelet count (/mm3)  T0  T1  T3  T5  T10 | 255 (220-324)  250 (212-297)  234 (196-304)  230 (197-289)  229 (196-272) | 211 (181-253)  203 (169-310)  184 (165-209)  221 (179-270)  196 (159-231) | 253 (208-316)  245 (209-297)  230 (193-295)  229 (197-285)  228 (192-265) |
| Free copper (ug/dL)  T0  T1  T3  T5  T10 | 10 (7-15)  0 (-8-11)  2 (0-5)  1 (-5.8-8)  2 (-4.1-9) | 14.4 (7.5-37)  -0.2 (-6-5)  6.4 (0-18)  3.7 (-2-6)  -1.2 (-5-2) | 11 (7-19)  -0.1 (-6-7)  2 (0-6)  2.4 (-5.8-8)  1 (-4.1-7.6) |
| Liver Stiffness (Kpas)  T0 (n=9) *  T1 (n=113)  T3 (n=41)  T5 (n=8)  Latest done (n=126) | 5.9 (4.4-6.4)  5.5 (4.7-7.1)  5.4 (4.6-6.9)  6.3 (4.8-10.4)  5.5 (4.7-6.8) | 25.7 (21.3-32.8)  11.8 (6.5-15.5)  7.8 (6.7-9.7)  6.8 (6.8-6.8)  8 (5.5-19) | 6.4 (5.5-21.3)  5.8 (4.9-8)  5.9 (4.6-7.6)  6.6 (5.3-8.9)  5.6 (4.8-7) |
